# Coronary and Cerebrovascular Events and Exacerbation of Existing Conditions After Laboratory‐Confirmed Influenza Infection Among US Veterans: A Self‐Controlled Case Series Study

**DOI:** 10.1111/irv.13304

**Published:** 2024-06-06

**Authors:** Caroline Korves, Nabin Neupane, Jeremy Smith, Yinong Young‐Xu, Robertus van Aalst, Salaheddin M. Mahmud, Matthew M. Loiacono

**Affiliations:** ^1^ Clinical Epidemiology Program Veterans Affairs Medical Center White River Junction Vermont USA; ^2^ PBM, Center for Medication Safety US Department of Veterans Affairs Hines Illinois USA; ^3^ Department of Health Services, Policy, and Practice Brown University School of Public Health Providence Rhode Island USA; ^4^ Global Medical Evidence Generation Sanofi Swiftwater Pennsylvania USA; ^5^ Vaccine and Drug Evaluation Centre, Department of Community Health Sciences University of Manitoba Winnipeg Manitoba Canada

**Keywords:** AMI, cerebrovascular, coronary, influenza, stroke, Veterans

## Abstract

**Background:**

Influenza may contribute to coronary/cerebrovascular events and exacerbate underlying conditions.

**Methods:**

We used self‐controlled case series (SCCS) design to analyze data from US Veterans ≥18 years with coronary/cerebrovascular or exacerbation event +/−1 year of lab‐confirmed influenza (LCI) during 2010–2018. We estimated the incidence ratio (IR) (95% CI) of the event for risk interval (Days 1–7 post‐LCI) versus control interval (all other times +/−1 year of LCI) with fixed‐effects conditional Poisson regression. We included biomarker data for mediation analysis.

**Results:**

We identified 3439 episodes with coronary/cerebrovascular‐related hospitalizations. IRs (95% CI) for LCI risk versus control interval were STEMI 0.6 (0.1, 4.4), NSTEMI 7.3 (5.8, 9.2), ischemic stroke 4.0 (3.0, 5.4), hemorrhagic stroke 6.2 (3.4, 11.5), and coronary spasm 1.3 (0.5, 3.0). IR significantly increased for NSTEMI and ischemic stroke among those ≥ 65 years. IR for NSTEMI and ischemic stroke dropped 26% and 10%, respectively, when white blood cell (WBC) and platelet count were considered. LCI was significantly associated with exacerbation of preexisting asthma, chronic obstructive pulmonary disease, and congestive heart failure.

**Conclusions:**

We found significant association between LCI and hospitalization for NSTEMI, ischemic stroke, and hemorrhagic stroke, the latter possibly due to unaccounted time‐varying confounding in SCCS design.

## 1 Introduction

Seasonal influenza presents a high burden to the United States in terms of morbidity and mortality. The Centers for Disease Control and Prevention (CDC) estimates that between 2010 and 2020, influenza resulted in 9–40 million illnesses and 12,000–52,000 deaths annually [1]. Although most individuals infected with influenza experience a self‐limited uncomplicated upper respiratory tract illness, more severe complications such as coronary or cerebrovascular events may follow the infection [2, 3]. Prior studies demonstrated an association between seasonal influenza activity and mortality due to cardiopulmonary causes [4]. Viral infections can lead to destabilization of atherosclerotic plaques, hypoxemia, and other mechanistic triggers for myocardial injury [5]. One study found nearly 12% of US adults hospitalized with laboratory‐confirmed influenza (LCI) had an acute cardiovascular event [2].

Understanding the relationship between LCI and a range of diseases can help guide health service planning during epidemics and may reinforce recommendation for vaccination [6]. Previous studies have shown a significant association with acute myocardial infarction and ischemic stroke in the week following LCI [6, 7, 8, 9, 10, 11]. We sought to extend this prior work by examining the association between LCI and additional coronary and cerebrovascular outcomes and exacerbation of underlying conditions, accounting for the role of mediating factors affecting inflammation and coagulation.

## 2 Methods

This study protocol was approved by the institutional review board of the Veterans Affairs (VA) Medical Center in White River Junction, VT, and was granted an exemption of consent.

### 2.1 Data Source

We used data from the Veterans Health Administration (VHA) of the Department of Veterans Affairs, the largest integrated healthcare system in the United States [12]. Data for this study were extracted from the VHA Corporate Data Warehouse (CDW) where we accessed electronic medical record and demographic information for VHA‐enrolled veterans. Each patient treated within VHA is assigned a unique identification number allowing longitudinal follow‐up across multiple locations. For veterans treated outside of VHA reimbursed by Medicare, Centers for Medicare & Medicaid Services (CMS) administrative claims data linked to VHA data in CDW, were included for analyses of individuals ≥ 65 years old.

### 2.2 Study Design

**FIGURE 1:**
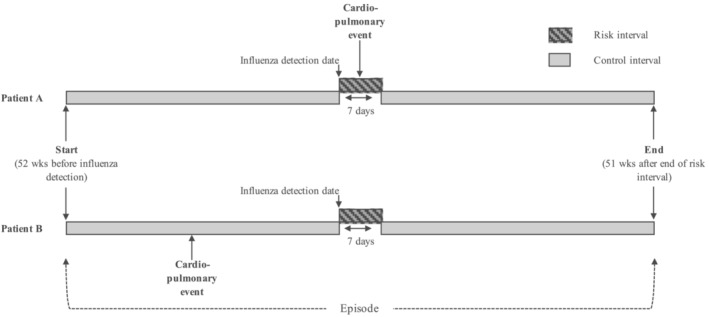
SCCS study design. An individual with an influenza infection who experienced a cardiopulmonary event during the 7‐day risk window (dark shading) or in the control window (light shading) could be included as a case.

Following prior work [7, 8], we used a self‐controlled case series (SCCS) design—only individuals who experienced an LCI and a coronary/cerebrovascular event or exacerbation during the observation period were included, eliminating time‐invariant confounding, as individuals act as their own controls. An episode was defined as an LCI and coronary/cerebrovascular event or exacerbation event within the year prior or following the LCI [7, 8]. The date of respiratory specimen collection served as the index date for defining LCI risk as the exact date of infection onset could not be determined. A period of 1 week after LCI was identified as the risk interval, and the control interval included all other periods of observation from 1 year prior to 1 year post‐LCI (Figure 1). The study period was defined as July 1, 2009, to June 30, 2019, during which it is possible an individual had more than one LCI and event. Each LCI episode contributed to the analysis.

Primary analyses focused solely on coronary/cerebrovascular events defined as hospitalization for acute myocardial infarction (AMI), ST‐elevation myocardial infarction (STEMI), non‐ST‐elevation myocardial infarction (NSTEMI), ischemic stroke, hemorrhagic stroke/major bleed, and unstable angina/coronary spasm identified by ICD‐9/10 codes (Table S1). Secondary analyses were restricted to individuals with either asthma, congestive heart failure (CHF), or chronic obstructive pulmonary disease (COPD) (Table S1), and exacerbation events were identified among those who had an LCI and hospitalization for their condition in either the year preceding or following LCI. In sensitivity analyses, risk intervals at Days 8–14, 15–22, 1–14, and 1–21 after LCI were evaluated. We also conducted an analysis with an induction interval of 7 days prior to LCI and excluded these days from the control period.

### 2.3 Patient Population

We included veterans ≥ 18 years old during the observation period, with a minimum of 12‐month enrolment in the database before the episode and ≥ 1 service record during the 12 months preceding the index date. All included veterans had LCI, including those from antigen and polymerase chain reaction (PCR) tests, during the period of July 1, 2010, to June 30, 2018, and, for primary analyses, were hospitalized for at least one coronary/cerebrovascular outcome event between July 1, 2009, and June 30, 2019, and within 1 year of LCI. The analyses for STEMI and NSTEMI were based on ICD‐10 codes due to the lack of specificity in their ICD‐9 codes. Thus, analyses for these episodes were limited to October 2015 and onwards as the Department of Health and Human Services mandated all Health Insurance Portability and Accountability Act covered entities transition to ICD‐10 codes on October 1, 2015 [13]. We excluded incidents where positive LCI specimens were obtained within 14 days after a previous positive specimen from the same patient (because we wanted to look at the time after onset of infection and assumed tests within 14 days were the same infection), and we excluded any coronary/cerebrovascular events that were transfers between hospitals or admissions within 30 days after a previous hospital discharge for the same severe event of interest as we were interested in looking at the first event of the care episode. Additionally, we excluded cases in which the positive influenza specimen was obtained during the hospitalization for the coronary/cerebrovascular outcome, as this would limit our ability to determine a temporal relationship between LCI and the coronary/cerebrovascular event; this exclusion also limited the possibility of enriching LCI at the time of the event that might otherwise occur due to routine workup of respiratory virus testing.

We collected patient demographic and clinical characteristic data from the 1‐year baseline period prior to the episode, such as record of the coronary/cerebrovascular event of interest and comorbidities. We also collected laboratory data for patients including influenza virus type (A untyped or identified by subtype, B, or other/unknown) and white blood cell (WBC) and platelet count during the risk period.

For secondary analyses where exacerbations of underlying conditions were evaluated, we restricted the population for each analysis to individuals with the underlying condition of interest and followed the same approach as the primary analysis to identify episodes.

### 2.4 Statistical Analysis

Using a fixed‐effects Poisson regression model, we estimated the incidence ratio (IR) and 95% CI for each coronary/cerebrovascular event or exacerbation event for risk versus control interval [7, 8, 14]. The association represents the presumed total effect of LCI on outcome. For coronary or cerebrovascular events found to be significantly associated with LCI, we conducted mediation analyses where WBC and platelet count during the LCI risk interval were the mediators of interest. Mediation analysis with these factors can explain potential mechanistic pathways by which LCI may raise the risk of coronary/cerebrovascular event. Elevated WBC and platelet count were selected because infection is a common cause of both and atherosclerotic‐plaque disruption and thrombosis are associated with coronary/cerebrovascular events [15, 16]. These laboratory measures are routinely collected for patients, unlike others such as troponin, which may be more frequently obtained for those at higher risk of the outcome of interest. For the mediation analyses, where the original model with the main effect of LCI is called Model 1, separate models were run where WBC at time of LCI (high, normal, low) was added to the original model (Model 2), platelet count at time of LCI (high, normal, low) was added to the original model (Model 3), and both WBC and platelet count were added to the original model (Model 4). The fixed‐effects conditional Poisson regression model was used to estimate the IR and confidence intervals, and the percent change in IR for the main effect of LCI from each model to the original model was estimated. The percent change represents the proportion of the association between LCI and the outcome that can be explained by the mediating variable. The normal range for WBC and platelet count were defined as 4500–11,000 WBCs per microliter and 150,000–400,000 platelets per microliter [17, 18]. If WBC or platelet count was not recorded for the risk interval, the value was assumed to be within normal range.

Time‐invariant confounders (e.g., age group, gender) need not be included with the SCCS study design and analysis because they remain constant in the risk and control interval. However, there are time‐invariant factors that may act as effect modifiers. Therefore, we conducted stratified analyses by characteristics that existed prior to the measured exposure of LCI and may have modified the risk of LCI on outcome and conducted a likelihood ratio tests (LRT) to compare models with and without an interaction term for LCI and the factor to assess whether there was no effect modification. The characteristics included age ≥ 65 years, influenza vaccination prior to the LCI, history of the coronary/cerebrovascular event, history of asthma, CHF, and COPD.

### 2.5 Post Hoc Analysis

In the primary analysis, we observed a significant association between LCI and hemorrhagic stroke. We hypothesized there may have been differential use of anticoagulation therapy near the time of LCI, which may have contributed to an elevation of hemorrhagic stroke risk. Although the SCCS study design adjusts for factors that are constant within an individual over time, it does not for factors that may change within the observation period. We therefore conducted an analysis adjusting for the time‐varying factor of anticoagulation therapy across the control and risk intervals. We also conducted post hoc sensitivity analyses and estimated E‐values to assess the strength of unmeasured confounding needed to explain the observed associations [19].

In all analyses, statistical tests were two‐tailed, and *p* values less than 0.05 indicated statistical significance. We used SAS software, Version 9.4, for analyses.

## 3 Results

Across the study period, we identified episodes of LCI with coronary/cerebrovascular events, including 2148 AMI, 167 STEMI, 1167 NSTEMI, 1212 ischemic stroke, 193 hemorrhagic stroke, and 415 unstable angina (Figure S1). The event categories for STEMI and NSTEMI were restricted to October 2015 and later when VHA switched to ICD‐10 codes; therefore, the number of AMI events is greater than the sum of STEMI and NSTEMI events (Table 1). Patient demographics were consistent across event episodes, with most included veterans being White (> 69%), non‐Hispanic (> 85%), male (> 94%), and urban dwellers (> 60%). The age of included veterans was also consistent, with 75% being over 65 years old (mean: 70 years) in all event categories except for unstable angina, which skewed younger. Most individuals (> 76%) had been vaccinated for influenza during the season at least 2 weeks prior to the LCI. Influenza virus type A was dominant accounting for approximately 65% of LCIs. Most included veterans had normal WBC (58%–64% across event categories) and normal platelet count (57%–68% across event categories) at time of LCI. Those with abnormal WBC trended consistently toward high WBC (20%–23% across event categories). Abnormal platelet counts were too low to be reported for STEMI and hemorrhagic stroke categories; however, across remaining event types, low platelet was observed for 32% and high platelet count for 3%.

**TABLE 1:** Patient characteristics and infection characteristics of the LCI and coronary/cerebrovascular event episodes.

	AMI^a^		STEMI		NSTEMI		Ischemic stroke		Hemorrhagic stroke/major bleed		Unstable angina/coronary spasm	
Number of episodes	2148	%	167	%	1167	%	1212	%	193	%	415	%
Age (years) at LCI, mean (standard deviation)	71.6 (11.1)		71.6 (11.3)		71.8 (10.74)		72.0 (10.9)		73.23 (12.3)		66.5 (10.7)	
Age (years) at LCI, *n* (%)												
< 65	521	24.3%	37	22.2%	259	22.2%	293	24.2%	41	21.2%	175	42.2%
65–74	861	40.1%	73	43.7%	497	42.6%	456	37.6%	63	32.6%	156	37.6%
75+	766	35.7%	57	34.1%	411	35.2%	463	38.2%	89	46.1%	84	20.2%
Sex												
Female	67	3.1%	NR	NR	29	2.5%	41	3.4%	NR	NR	21	5.1%
Male	2081	96.9%	NR	NR	1138	97.5%	1171	96.6%	NR	NR	394	94.9%
Race/ethnicity, *n* (%)												
Asian	13	0.6%	NR	NR	NR	NR	NR	NR	NR	NR	NR	NR
Black	446	20.8%	31	18.6%	259	22.2%	270	22.3%	37	19.2%	74	17.8%
White	1538	71.6%	116	69.5%	818	70.1%	856	70.6%	138	71.5%	313	75.4%
Other	35	1.6%	NR	NR	NR	NR	NR	NR	NR	NR	NR	NR
Unknown/missing	116	5.4%	11	6.6%	64	5.5%	64	5.3%	14	7.3%	19	4.6%
Hispanic, *n* (%)												
Yes	99	4.6%	NR	NR	58	5.0%	62	5.1%	15	7.8%	15	3.6%
No/missing	2049	95.4%	NR	NR	1109	95.0%	1150	94.9%	178	92.2%	400	96.4%
Rurality, *n* (%)												
Rural	589	27.4%	30	18.0%	314	26.1%	366	30.2%	64	33.2%	149	35.9%
Urban	1548	72.1%	137	82.0%	845	72.4%	844	69.6%	129	66.8%	263	63.4%
Missing	11	0.5%	0	0.0%	8	0.7%	2	0.2%	0	0.0%	3	0.7%
Prior specific cardiopulmonary hospitalization, *n* (%)	150	7.0%	NR	NR	60	5.1%	104	8.6%	NR	NR	31	7.5%
Comorbidities, *n* (%)												
Asthma	148	6.9%	13	7.8%	84	7.2%	55	4.5%	12	6.2%	33	8.0%
CHF	642	29.9%	35	21.0%	375	32.1%	294	24.3%	43	22.3%	97	23.4%
COPD	720	33.5%	47	28.1%	415	35.6%	357	29.5%	52	26.9%	146	35.2%
Diabetes	1101	51.3%	82	49.1%	616	52.8%	592	48.8%	82	42.5%	192	46.3%
Hypertension	1757	81.8%	124	74.3%	975	83.5%	994	82.0%	151	78.2%	329	79.3%
Hyperlipidemia	1419	66.1%	101	60.5%	794	68.0%	800	66.0%	118	61.1%	289	69.6%
Virus type, *n* (%)												
A	1356	63.1%	107	64.1%	772	66.2%	781	64.4%	130	67.4%	255	61.4%
B	499	23.2%	49	29.3%	288	24.7%	276	22.8%	43	22.3%	73	17.6%
Other/unknown	293	13.6%	11	6.6%	107	9.2%	155	12.8%	20	10.4%	87	21.0%
White blood cell count, *n* (%)												
High	511	23.8%	34	20.4%	267	22.9%	289	23.8%	45	23.3%	94	22.7%
Low	378	17.6%	25	15.0%	209	17.9%	211	17.4%	34	17.6%	64	15.4%
Normal	1259	58.6%	108	64.7%	691	59.2%	712	58.7%	114	59.1%	257	61.9%
Platelet count, *n* (%)												
High	70	3.3%	NR	NR	43	3.7%	36	3.0%	NR	NR	12	2.9%
Low	692	32.2%	NR	NR	376	32.2%	397	32.8%	NR	NR	129	31.1%
Normal	1386	64.5%	114	68.3%	748	64.1%	779	64.3%	111	57.5%	274	66.0%
Vaccinated for influenza, *n* (%)^b^	1774	82.6%	128	76.6%	989	84.7%	970	80.0%	154	79.8%	332	80.0%
Pneumonia within 7 days of LCI, *n* (%)	266	12.4%	NR	NR	156	13.4%	133	11.0%	22	11.4%	41	9.9%

Abbreviations: AMI, acute myocardial infarction; CHF, congestive heart failure; COPD, chronic obstructive pulmonary disease; LCI, laboratory‐confirmed influenza; NR, not reported (exact number cannot be reported for privacy reasons); NSTEMI, non‐ST‐elevation myocardial infarction; STEMI, ST‐elevation myocardial infarction; WBC, white blood cell.

^a^ The event categories for STEMI and NSTEMI were restricted to October 2015 and later when VHA switched to ICD‐10 codes; therefore, the number of AMI events is greater than the sum of STEMI and NSTEMI events.

^b^ Received influenza vaccination during season of the episode's LCI and at least 2 weeks prior to LCI. An influenza season is from July 1, year a (e.g., 2010) until June 30, year a + 1 (e.g., 2011).

The IRs of outcomes for LCI risk interval versus control period, overall, and restricted to those 65 years old and over appear in Table 2. There were significant associations (IR [95% CI]) with LCI for AMI (7.0 [5.9, 8.3]), NSTEMI (7.3 [5.8, 9.2]), ischemic stroke (4.0 [3.0, 5.4]) and hemorrhagic stroke (6.2 [3.4, 11.5]).

**TABLE 2:** Risk of coronary/cerebrovascular event in the week following LCI.

	Number episodes	Episodes with event during risk interval (Days 1–7 after LCI)	Episodes with event during control interval	IR (95% CI)
LCI July 1, 2010, to June 30, 2018, cardiopulmonary event identified by ICD‐9 or ICD‐10 codes				
AMI	2148	136	2012	7.0 (5.9, 8.3)
Ischemic stroke	1212	45	1167	4.0 (3.0, 5.4)
Hemorrhagic stroke/major bleed	193	11	182	6.2 (3.4, 11.5)
Unstable angina/coronary spasm	415	NR	NR	1.3 (0.5, 3.0)
LCI July 1, 2010, to June 30, 2018, cardiopulmonary event identified by ICD‐9 or ICD‐10 codes, restricted to episodes among individuals 65 + years old				
AMI	1627	120	1507	8.2 (6.8, 9.9)
Ischemic stroke	919	40	879	4.7 (3.4, 6.5)
Hemorrhagic stroke/major bleed	152	11	141	8.1 (4.4, 14.9)
Unstable angina/coronary spasm	240	NR	NR	1.8 (0.6, 4.7)
Cardiopulmonary event identified by ICD‐10 codes only				
STEMI	167	NR	NR	0.6 (0.1, 4.4)
NSTEMI	1167	77	1090	7.3 (5.8, 9.2)
Cardiopulmonary event identified by ICD‐10 codes only, restricted to episodes among individuals 65 + years old				
STEMI	130	0	130	0
NSTEMI	908	70	838	8.6 (6.8, 11.0)

Abbreviations: AMI, acute myocardial infarction; IR, incidence ratio; LCI, laboratory‐confirmed influenza; NR, not reported (because observation is <11 and exact number cannot be reported for privacy reasons); NSTEMI, non‐ST‐elevation myocardial infarction; STEMI, ST‐elevation myocardial infarction.

Mediation analysis was performed for each coronary/cerebrovascular event category where a significant association with LCI was found (Table 3). The IR for NSTEMI dropped from 7.3 to 5.8 (20%) when WBC was included in the model, from 7.3 to 6.8 (7%) when platelet count was included, and from 7.3 to 5.4 (26%) when both were included. For ischemic stroke, the IR increased by 2% with the inclusion of WBC, decreased by 8% when platelet count was included, and decreased by 10% when both were included. For hemorrhagic stroke, the IR decreased by 8% with WBC included, increased by 30% with platelet count included, and increased by 13% with both.

**TABLE 3:** Estimation of the mediating effects of white blood cell count and platelet count on the association between LCI and coronary/cerebrovascular events.

	Model 1	Model 2	Model 3	Model 4
AMI				
LCI	7 (5.9, 8.3)	6.2 (4.9, 7.9)	6.8 (5.4, 8.4)	5.9 (4.5, 7.7)
WBC high		1.7 (1.2, 2.5)		1.8 (1.2, 2.7)
WBC low		0.8 (0.5, 1.4)		0.7 (0.4, 1.3)
Platelets high			0.4 (0.1, 1.9)	0.3 (0.1, 1.5)
Platelets low			1.2 (0.8, 1.7)	1.3 (0.9, 1.8)
Change in LCI		11%	3%	16%
LRT *p* (vs. Model 1)		< 0.01	0.28	< 0.01
NSTEMI				
LCI	7.3 (5.8, 9.2)	5.8 (4.2, 8.1)	6.8 (5, 9.1)	5.4 (3.8, 7.8)
WBC high		2.3 (1.4, 3.8)		2.4 (1.5, 4)
WBC low		0.8 (0.4, 1.7)		0.7 (0.3, 1.5)
Platelets high			0.7 (0.2, 3.2)	0.5 (0.1, 2.1)
Platelets low			1.3 (0.8, 2.1)	1.3 (0.8, 2.2)
Change in LCI		20%	7%	26%
LRT *p*		< 0.01	0.53	< 0.01
Ischemic stroke				
LCI	4 (3, 5.4)	4.1 (2.8, 6)	3.7 (2.5, 5.5)	3.6 (2.3, 5.6)
WBC high		1.4 (0.7, 2.6)		1.4 (0.7, 2.8)
WBC low		0.4 (0.1, 1.2)		0.3 (0.1, 1.1)
Platelets high			0.8 (0.1, 6)	0.6 (0.1, 4.9)
Platelets low			1.2 (0.7, 2.3)	1.5 (0.8, 2.9)
Change in LCI		2%	8%	10%
LRT *p* (vs. Model 1)		0.06	0.75	0.11
Hemorrhagic stroke/major bleed				
LCI	6.2 (3.4, 11.5)	5.7 (2.5, 13.1)	8 (3.9, 16.5)	7 (2.9, 16.9)
WBC high		1.8 (0.5, 6.5)		1.7 (0.4, 6.6)
WBC low		0.5 (0.1, 4.7)		0.7 (0.1, 6.8)
Platelets high			1.8 (0.2, 16.9)	1.6 (0.2, 15.3)
Platelets low			0.4 (0.1, 1.7)	0.4 (0.1, 1.9)
Change in LCI		8%	30%	13%
LRT *p*		0.51	0.29	0.51

Abbreviations: AMI, acute myocardial infarction; LCI, laboratory‐confirmed influenza; NSTEMI, non‐ST‐elevation myocardial infarction; WBC, white blood cell count.

Stratified SCCS analysis results appear in Table S2. The IR for NSTEMI and ischemic stroke for LCI versus control interval were significantly higher for those 65 years old and over.

Secondary analysis examined the association between LCI and exacerbation resulting in hospitalization of existing conditions in subpopulations with asthma, COPD, and CHF during risk versus control interval (Table 4). The increased risk following LCI was seen in all three subpopulations (IR [95% CI]: 10.6 (8.0, 14.0) for asthma, 12.5 [11.1, 14.0] for COPD, 7.2 [6.0, 8.7] for CHF).

**TABLE 4:** Risk of severe exacerbation event associated with LCI among individuals with asthma, COPD, and CHF.

	Number episodes	Episodes with event during risk interval (Days 1–7 after LCI)	Episodes with event during control interval	IR (95%CI)
LCI July 1, 2010, to June 30, 2018, severe exacerbation event identified by ICD‐9 or ICD‐10 codes				
Asthma	584	54	530	10.6 (8.0, 14.0)
COPD	2893	311	2582	12.5 (11.1, 14.0)
CHF	1880	123	1757	7.2 (6.0, 8.7)

Abbreviations: CHF, congestive heart failure; COPD, chronic obstructive pulmonary disease; IR, incidence ratio; LCI, laboratory‐confirmed influenza.

### 3.1 Sensitivity Analysis

When the risk interval was delayed from Days 1–7 post‐LCI to Days 8–14, the risk from LCI for AMI, NSTEMI, and ischemic stroke persisted but was dampened; the risk for AMI and ischemic stroke on Days 15–21was even lower, and there was no longer an association between LCI and NSTEMI at that point (Table S3). A similar dampening of risk of LCI on AMI, NSTEMI, and ischemic stroke was observed when evaluating risk periods of Days 1–14 and 1–21 versus Days 1–7 post‐LCI. Including an induction period had little effect on the IRs.

### 3.2 Post Hoc Analysis

Notably, the IR for LCI versus control interval for hemorrhagic stroke was 6.2 (3.4, 11.5). Because the biological plausibility of LCI affecting risk of hemorrhagic stroke is not as strong as for the other event categories, we considered whether there was some time‐variant confounding, which would not be adjusted for with the planned SCCS study design. With adjustment for the time‐varying factor of anticoagulation therapy the IR decreased to 1.9 (1.3, 2.7). Based on the calculation of the E‐value, if unmeasured confounding remained after adjustment and accounted for the non‐null association, the unmeasured confounding would need to be associated with LCI and hemorrhagic stroke by a factor of 3.2 each; weaker confounding could not account for the observed association. For the non‐null associations observed in the main analysis between LCI and NSTEMI (and LCI and ischemic stroke), unmeasured confounding would need to be associated with LCI and NSTEMI by a factor of 14.1 each (and 7.5 for LCI and stroke) for unmeasured confounding to explain the observed association.

## 4 Discussion

We found the risk of any AMI, NSTEMI, ischemic stroke, and hemorrhagic stroke was four to seven times greater in the week following LCI compared with the year prior to and after infection. The risk of NSTEMI was significantly greater for those 65 years and over. The risk of ischemic stroke was significantly greater for those 65 years and over as well. Whereas the risk from LCI for NSTEMI and ischemic stroke was lower for those under 65 years old, the increased risk remained significant. Our findings emphasize the substantial associated risk of coronary/cerebrovascular morbidity following influenza infection and highlight the importance of both high coverage and effectiveness of vaccination for all individuals and particularly for those 65 years and over.

Whereas prior studies have found the risk of AMI increases following LCI, with IRs ranging from 5.39 to 17.5, this study separated STEMI from NSTEMI and found a significant association for NSTEMI within the range of IRs previously observed for AMI [6, 7, 8, 9, 10]. The number of episodes with NSTEMI versus STEMI was about seven times greater in the current study, aligning with the fact that approximately 70% of cases of acute coronary syndrome are NSTEMI [20]. Furthermore, viral infection, including influenza, can cause inflammation in the heart, leading to a rise in the protein troponin used in the diagnosis of NSTEMI [21, 22]. A prior study reported a significantly higher risk for STEMI in the first 3 days following a care consultation for acute respiratory infection; however, that study included infections additional to influenza and did not have laboratory confirmation data to identify exposure [10]. The risk of ischemic stroke following LCI has not been studied extensively. Our new findings are consistent with a small study showing the IR for ischemic stroke was 10.3 (4.2, 25.4) and 6.5 (2.4, 17.7) for Days 1–3 and Days 4–7 past influenza symptom onset and another study showing significant risk for Days 1–3, 8–14, and 15–28 after testing, with an IR reaching 8.13 (1.98, 33.3) [6, 9].

Unlike the increased risk of AMI or ischemic stroke following LCI, which can be explained by the pro‐thrombotic state it induces [23], the increased risk of hemorrhagic stroke did not have the same level of biologic plausibility. We believe this association might be confounded by anticoagulant use among hospitalized patients to prevent thrombosis [24], as evidenced by the IR for hemorrhagic stroke dropping from 6.2 to 1.9 once anticoagulant use was adjusted for in post hoc analysis. The E‐value estimate indicates that for remaining confounding to explain the association observed in the adjusted analysis, the confounding would need to be associated with both LCI and hemorrhagic stroke by a factor of at least 3.2. When looking further at the distribution of hemorrhagic stroke times relative to LCI, it appears that one and a half times as many occur in the control period 2–12 months after LCI versus 2–12 months pre‐LCI, which could indicate some remaining time‐variant confounding. Further investigation of this association and impact of treatments is important as it may have implications for management of influenza and balancing prevention of ischemic stroke with risk of major bleed.

The analysis of mediating factors for the association between LCI and NSTEMI indicated that WBC and platelet counts, but more so WBC, may be mediating factors. For the analysis of the association between LCI and ischemic stroke, both may be mediating factors, but platelet count more so. Our results indicate that only 26% of the association between LCI and NSTEMI could be explained by WBC and platelet count and 10% of the association between LCI and ischemic stroke. Our finding for NSTEMI is only slightly higher than the AMI association explained by WBC and platelet count previously reported at 20% [8].

Importantly, findings from the current study show that the risk of exacerbation of preexisting diagnoses of asthma, COPD, and CHF is high, up to 12‐fold higher for CHF. A prior study showed a significant risk of CHF following LCI but did not focus on exacerbation of a preexisting condition [25]. Given that approximately one in 13 persons in the United States has asthma and 6.4% have COPD, these findings are clinically significant and reinforce the importance of influenza vaccination among these risk groups [26, 27].

This study has notable strengths including the use of laboratory data drawn from the vast VHA to identify influenza and biomarkers of interest. Because almost all Medicare‐eligible veterans have Medicare Part A, including Medicare data captured hospital utilization comprehensively for the 65+ population, reducing data loss of events outside the VHA. One potential limitation of this study is the overestimation of event rates due to LCI for the non‐Medicare‐eligible population if these individuals are more likely to seek care at the VA after influenza testing at VA versus other times in the 2‐year control period. Additionally, the SCCS design controls for time‐invariant confounding but not time‐varying confounding, which remains a possibility given the 2‐year control period. Also, if patients at higher risk of coronary/cerebrovascular events were more likely to be tested for flu, this could have overestimated the association between LCI and these events. Some laboratory values were sparse, and we considered WBC and platelet counts measured during the Days 1–7 post‐LCI risk period regardless of whether the counts were taken prior or following the coronary/cerebrovascular event. Furthermore, the very severe events resulting in death before hospitalization would not be included, though such bias should be non‐differential. Finally, the VHA patient population is largely male and older, limiting the generalizability of the study findings.

Our study highlights the need to protect individuals from influenza to guard against increased risk of coronary and cerebrovascular events and exacerbation of underlying conditions. Elevation in WBC and platelet count may mediate some of the observed associations and be useful measures to monitor among individuals with influenza. Future studies may further explore how best to manage and treat inflammation and coagulation following influenza without raising the risk of unwanted treatment‐related events.

## Author Contributions

**Caroline Korves:** investigation, methodology, project administration, supervision, writing–original draft, writing–review and editing. **Nabin Neupane:** formal analysis, writing–original draft, writing–review and editing. **Jeremy Smith:** formal analysis, investigation, writing–original draft, writing–review and editing. **Yinong Young‐Xu:** conceptualization, funding acquisition, methodology, writing–original draft, writing–review and editing. **Robertus van Aalst:** conceptualization, methodology, writing–original draft, writing–review and editing. **Salaheddin M. Mahmud:** methodology, writing–original draft, writing–review and editing. **Matthew M. Loiacono:** methodology, writing–original draft, writing–review and editing.

## Conflicts of Interest

C.K., N.N., J.S., and Y.Y.X. have worked with or at VERANNE that received funding from Sanofi for this project; they reported funding from Pfizer for other research projects outside the submitted work. R.vA. and M.M.L. are full‐time employees of Sanofi, a company that makes influenza vaccines, and may hold shares/stocks in the company. S.M.M. has received unrestricted research grants from GlaxoSmithKline, Merck, Pfizer, Sanofi, and Roche‐Assurex. S.M.M. has received fees as a consultant and advisory board member for GlaxoSmithKline, Merck, Sanofi, and Seqirus. S.M.M.'s work is supported, in part, by funding from the Canada Research Chairs Program.

### Peer Review

The peer review history for this article is available at https://www.webofscience.com/api/gateway/wos/peer‐review/10.1111/irv.13304.

## Supporting information


**Table S1.** ICD‐9/10 codes to identify coronary/cerebrovascular events.

**Figure S1.** Identification of episodes of LCI with coronary/cerebrovascular events. Legend: Some LCI had more than one coronary/cerebrovascular event within +/−1 year, so the sum of LCI and coronary/cerebrovascular events is greater than 3569. Abbreviations: AMI, acute myocardial infarction; LCI, laboratory‐confirmed influenza; NSTEMI, non‐ST‐elevation myocardial infarction; STEMI, ST‐elevation myocardial infarction.

**Table S2.** Risk of coronary/cerebrovascular event associated with LCI, stratified SCCS.

**Table S3.** Risk of coronary and cerebrovascular event associated with LCI, sensitivity analysis of risk period.

## Data Availability

The US Department of Veterans Affairs (VA) places restrictions on access to veterans' healthcare data, which includes both identifying data and sensitive patient information. The analytic data sets used for this study are not permitted to be shared. This limitation is consistent with other studies based on VA data. However, VA data are made freely available to researchers behind the VA firewall with an approved VA study protocol. For more information, please visit https://www.virec.research.va.gov.
