# Supplementary material for: Coronary and Cerebrovascular Events and Exacerbation of Existing Conditions After Laboratory‐Confirmed Influenza Infection Among US Veterans: A Self‐Controlled Case Series Study
Source: Influenza Other Respir Viruses. 2024 Jun 6;18(6):e13304. doi: 10.1111/irv.13304 (PMC11157146; doi:10.1111/irv.13304)
Supplement: Supplementary file 1 — Table S1. ICD‐9/10 codes to identify coronary/cerebrovascular events. [file IRV-18-e13304-s004.docx]

**Supplemental**

**Appendix Table 1.** ICD-9/10 codes to identify coronary/cerebrovascular events

| **ST segment elevation myocardial infarction (STEMI)** | |
| --- | --- |
| **ICD-10 code** | **Description** |
| I21.0 | ST elevation (STEMI) myocardial infarction of anterior wall |
| I21.01 | ST elevation (STEMI) myocardial infarction involving left main coronary artery |
| I21.02 | ST elevation (STEMI) myocardial infarction involving left anterior descending coronary artery |
| I21.09 | ST elevation (STEMI) myocardial infarction involving other coronary artery of anterior wall |
| I21.1 | ST elevation (STEMI) myocardial infarction of inferior wall |
| [I21.11](https://www.icd10data.com/ICD10CM/Codes/I00-I99/I20-I25/I21-/I21.11) | ST elevation (STEMI) myocardial infarction involving right coronary artery |
| [I21.19](https://www.icd10data.com/ICD10CM/Codes/I00-I99/I20-I25/I21-/I21.19) | ST elevation (STEMI) myocardial infarction involving other coronary artery of inferior wall |
| I21.2 | ST elevation (STEMI) myocardial infarction of other sites |
| I21.21 | ST elevation (STEMI) myocardial infarction involving left circumflex coronary artery |
| I21.29 | ST elevation (STEMI) myocardial infarction involving other sites |
| I21.3 | ST elevation (STEMI) myocardial infarction of unspecified site |
| I22.0 | Subsequent ST elevation (STEMI) myocardial infarction of anterior wall |
| I22.1 | Subsequent ST elevation (STEMI) myocardial infarction of inferior wall |
| I22.8 | Subsequent ST elevation (STEMI) myocardial infarction of other sites |
| I22.9 | Subsequent ST elevation (STEMI) myocardial infarction of unspecified site |
|  |  |
| **Non-ST segment elevation myocardial infarction (NSTEMI)** | |
| **ICD-10 code** | **Description** |
| I21.4 | Non-ST elevation (NSTEMI) myocardial infarction |
| I22.2 | Subsequent non-ST elevation (NSTEMI) myocardial infarction |
|  |  |
| **Any AMI** | |
| **ICD-9 code** | **Description** |
| 410 | Acute myocardial infarction |
| 410.1 | Acute myocardial infarction of other anterior wall |
| 410.2 | Acute myocardial infarction of inferolateral wall |
| 410.3 | Acute myocardial infarction of inferoposterior wall |
| 410.4 | Acute myocardial infarction of other inferior wall |
| 410.5 | Acute myocardial infarction of other lateral wall |
| 410.6 | True posterior wall infarction |
| 410.7 | Subendocardial infarction |
| 410.8 | Acute myocardial infarction of other specified sites |
| 410.9 | Acute myocardial infarction of unspecified site |
| **ICD-10 code** |  |
| I21.0 | ST elevation (STEMI) myocardial infarction of anterior wall |
| I21.01 | ST elevation (STEMI) myocardial infarction involving left main coronary artery |
| I21.02 | ST elevation (STEMI) myocardial infarction involving left anterior descending coronary artery |
| I21.09 | ST elevation (STEMI) myocardial infarction involving other coronary artery of anterior wall |
| I21.1 | ST elevation (STEMI) myocardial infarction of inferior wall |
| [I21.11](https://www.icd10data.com/ICD10CM/Codes/I00-I99/I20-I25/I21-/I21.11) | ST elevation (STEMI) myocardial infarction involving right coronary artery |
| [I21.19](https://www.icd10data.com/ICD10CM/Codes/I00-I99/I20-I25/I21-/I21.19) | ST elevation (STEMI) myocardial infarction involving other coronary artery of inferior wall |
| I21.2 | ST elevation (STEMI) myocardial infarction of other sites |
| I21.21 | ST elevation (STEMI) myocardial infarction involving left circumflex coronary artery |
| I21.29 | ST elevation (STEMI) myocardial infarction involving other sites |
| I21.3 | ST elevation (STEMI) myocardial infarction of unspecified site |
| I21.4 | Non-ST elevation (NSTEMI) myocardial infarction |
| I21.9 | Acute myocardial infarction, unspecified |
| I21.A | Other type of myocardial infarction |
| I22.0 | Subsequent ST elevation (STEMI) myocardial infarction of anterior wall |
| I22.1 | Subsequent ST elevation (STEMI) myocardial infarction of inferior wall |
| I22.2 | Subsequent non-ST elevation (NSTEMI) myocardial infarction |
| I22.8 | Subsequent ST elevation (STEMI) myocardial infarction of other sites |
| I22.9 | Subsequent ST elevation (STEMI) myocardial infarction of unspecified site |
|  |  |
| **Coronary spasm, or unstable angina** | |
| **ICD-9 code** | **Description** |
| 411.1 | Intermediate coronary syndrome |
| **ICD-10 code** |  |
| I20.0 | Unstable angina |
|  |  |
| **Ischemic stroke (i.e., clots)** | |
| **ICD-9 code** | **Description** |
| 433 | Occlusion and stenosis of precerebral arteries |
| 434 | Occlusion of cerebral arteries |
| **ICD-10 code** |  |
| I63 | Cerebral infarction |
|  |  |
| **Hemorrhagic stroke (i.e., major bleeds)** | |
| **ICD-9 code** | **Description** |
| 430 | Subarachnoid hemorrhage |
| 431 | Intracerebral hemorrhage |
| 432.x | Other and unspecified intracranial hemorrhage |
| **ICD-10 code** |  |
| I60 | Nontraumatic subarachnoid hemorrhage |
| I61 | Nontraumatic intracerebral hemorrhage |
| I62.9 | Nontraumatic intracranial hemorrhage, unspecified |
