# Supplementary figures and images for: Coronary and Cerebrovascular Events and Exacerbation of Existing Conditions After Laboratory‐Confirmed Influenza Infection Among US Veterans: A Self‐Controlled Case Series Study
Source: Influenza Other Respir Viruses. 2024 Jun 6;18(6):e13304. doi: 10.1111/irv.13304 (PMC11157146; doi:10.1111/irv.13304)

**Appendix Figure 1.** Identification of episodes of LCI with coronary/cerebrovascular events


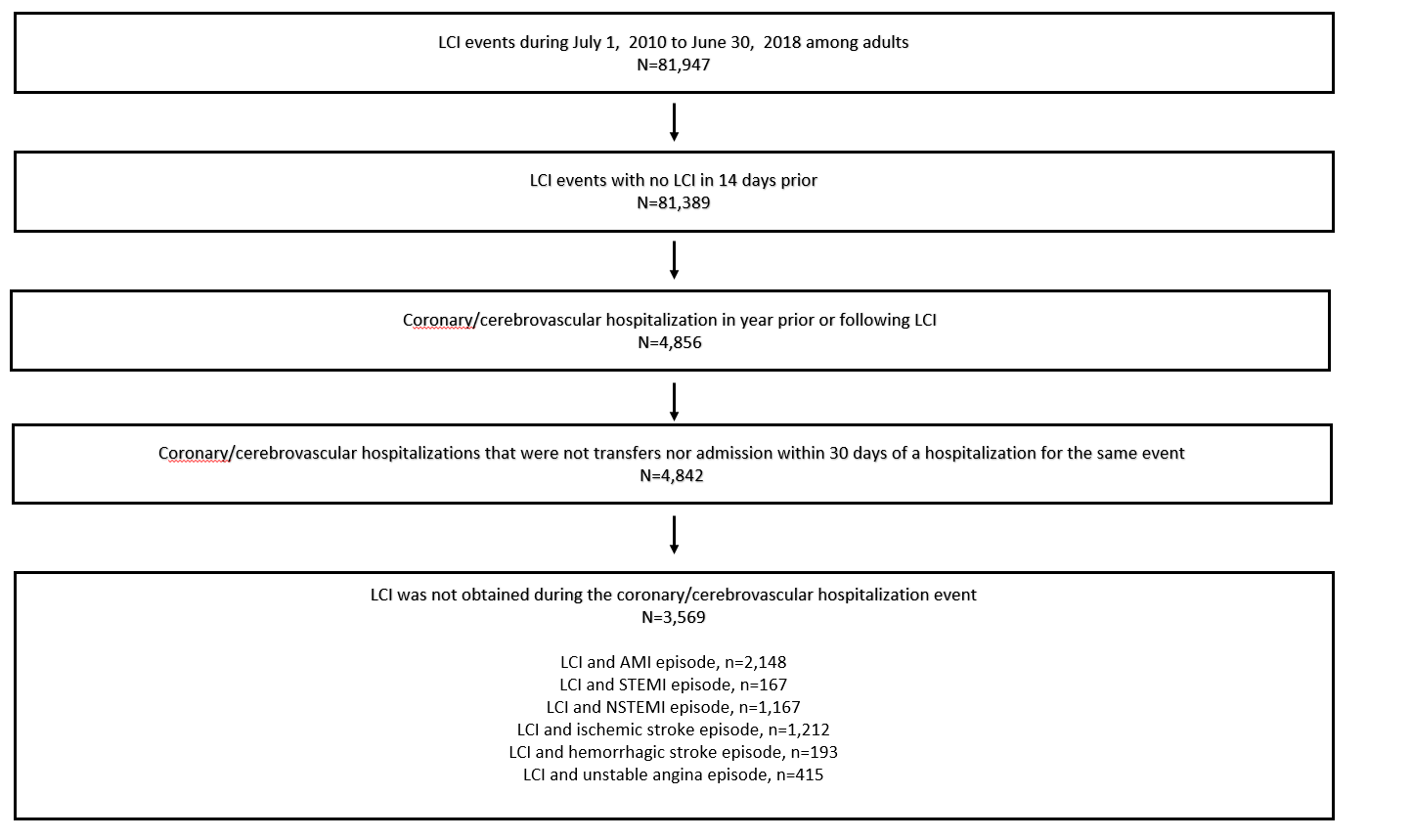

Supplement: Supplementary file 2 — Figure S1. Identification of episodes of LCI with coronary/cerebrovascular events. Legend: Some LCI had more than one coronary/cerebrovascular event within +/−1 year, so the sum of LCI and coronary/cerebrovascular events is greater than 3569. Abbreviations: AMI, acute myocardial infarction; LCI, laboratory‐confirmed influenza; NSTEMI, non‐ST‐elevation myocardial infarction; STEMI, ST‐elevation myocardial infarction. [file IRV-18-e13304-s003.docx]
