# Supplementary material for: Coronary and Cerebrovascular Events and Exacerbation of Existing Conditions After Laboratory‐Confirmed Influenza Infection Among US Veterans: A Self‐Controlled Case Series Study
Source: Influenza Other Respir Viruses. 2024 Jun 6;18(6):e13304. doi: 10.1111/irv.13304 (PMC11157146; doi:10.1111/irv.13304)
Supplement: Supplementary file 3 — Table S2. Risk of coronary/cerebrovascular event associated with LCI, stratified SCCS. [file IRV-18-e13304-s002.docx]

**Appendix Table 2. Risk of coronary/cerebrovascular event associated with LCI, stratified SCCS**

|  | AMI | | STEMI | | NSTEMI | | Ischemic stroke | | Hemorrhagic stroke/ major bleed | | Unstable angina/ coronary spasm | |
| --- | --- | --- | --- | --- | --- | --- | --- | --- | --- | --- | --- | --- |
|  | IR(95%CI) | p-value from LRT for interaction | IR(95%CI) | p-value from LRT for interaction | IR(95%CI) | p-value from LRT for interaction | IR(95%CI) | p-value from LRT for interaction | IR(95%CI) | p-value from LRT for interaction | IR(95%CI) | p-value from LRT for interaction |
|  |  |  |  |  |  |  |  |  |  |  |  |  |
| Age |  |  |  |  |  |  |  |  |  |  |  |  |
| <65 years | 3.3 (2,5.4) |  | 2.9 (0.4,20.9) |  | 2.9 (1.4,6.1) |  | 1.8 (0.7,4.3) |  | 0 (0, not estimable) |  | 0.6 (0.1,4.2) |  |
| ≥65 years | 8.2 (2.9,23) | <0.01 | 0 (0, non estimable) | 0.08 | 8.6 (1.9,40.3) | <0.01 | 4.7 (0.8,29.1) | 0.02 | 8.0 (0, not estimable) | 0.02 | 1.8 (0,113.3) | 0.29 |
|  |  |  |  |  |  |  |  |  |  |  |  |  |
| Influenza vaccination† | |  |  |  |  |  |  |  |  |  |  |  |
| No | 7.7 (5.2,11.5) |  | 0 (0, non estimable) |  | 6.1 (3.2,11.6) |  | 4.5 (2.4,8.4) |  | 5.6 (1.3,23.2) |  | 1.3 (0.2,9) |  |
| Yes | 6.8 (3,15.9) | 0.60 | 0.8 (0, non estimable) | 0.46 | 7.5 (2,28.2) | 0.56 | 3.9 (1,14.9) | 0.71 | 6.4 (0.3,128.4) | 0.86 | 1.3 (0,82.3) | 0.99 |
|  |  |  |  |  |  |  |  |  |  |  |  |  |
| Asthma history^‡^ | |  |  |  |  |  |  |  |  |  |  |  |
| No | 7 (5.9,8.4) |  | 0.7 (0.1,4.8) |  | 7.4 (5.8,9.4) |  | 3.8 (2.8,5.2) |  | 5.4 (2.8,10.6) |  | 1.4 (0.6,3.3) |  |
| Yes | 6.7 (2.8,16.2) | 0.91 | 0 (0, non estimable) | 0.69 | 6.5 (2,21.1) | 0.80 | 8.1 (2,32.1) | 0.20 | 20.7 (2,212.3) | 0.16 | 0 (0, not estimable) | 0.36 |
|  |  |  |  |  |  |  |  |  |  |  |  |  |
| COPD history^‡^ | |  |  |  |  |  |  |  |  |  |  |  |
| No | 7 (5.7,8.7) |  | 0.9 (0.1,6.2) |  | 7.7 (5.8,10.2) |  | 3.8 (2.6,5.4) |  | 4.6 (2,10.4) |  | 1.6 (0.6,4.2) |  |
| Yes | 6.9 (3.9,12.3) | 0.91 | 0 (0, non estimable) | 0.42 | 6.6 (3.1,14.4) | 0.55 | 4.5 (1.7,12.3) | 0.56 | 11 (1.4,85.4) | 0.18 | 0.7 (0,17.3) | 0.45 |
|  |  |  |  |  |  |  |  |  |  |  |  |  |
| CHF history^‡^ | |  |  |  |  |  |  |  |  |  |  |  |
| No | 7.8 (6.4,9.6) |  | 0.8 (0.1,5.6) |  | 8.2 (6.3,10.7) |  | 3.9 (2.7,5.5) |  | 5.1 (2.4,10.8) |  | 1.3 (0.5,3.5) |  |
| Yes | 5.1 (2.7,9.4) | 0.03 | 0 (0, non estimable) | 0.49 | 5.5 (2.5,12.3) | 0.14 | 4.4 (1.6,12.2) | 0.71 | 10.6 (1.4,81.2) | 0.27 | 1.1 (0,26.1) | 0.85 |
|  |  |  |  |  |  |  |  |  |  |  |  |  |
| Cardiopulmonary event history^‡, §^ | | |  |  |  |  |  |  |  |  |  |  |
| No | 7.1 (5.9,8.5) |  | 0.6 (0.1,4.5) |  | 7.2 (5.7,9.1) |  | 4 (2.9,5.4) |  | 6.4 (3.5,11.8) |  | 1.4 (0.6,3.3) |  |
| Yes | 6 (2.4,14.9) | 0.65 | 0 (0, non estimable) | 0.91 | 9.6 (2.9,31.3) | 0.57 | 4.2 (1.1,16.2) | 0.93 | 0 (0, not estimable) | 0.44 | 0 (0, not estimable) | 0.38 |
| **Abbreviations**: AMI, acute myocardial infarction; CHF, congestive heart failure; COPD, chronic obstructive pulmonary disease; IR, incidence ratio; LCI, laboratory-confirmed influenza; NSTEMI, non-ST-elevation myocardial infarction; SCCS, self-controlled case series; STEMI, ST-elevation myocardial infarction. | | | | | | | | | | | | |
|  |  |  |  |  |  |  |  |  |  |  |  |  |
| **Notes**: ^†^Received influenza vaccination during season of the episode's LCI and at least 2 weeks prior to LCI. An influenza season is from July 1, year a (e.g., 2010) until June 30, year a+1 (e.g., 2011). ^‡^For history of disease/ event, 2 outpatient or 1 inpatient visit with ICD-9 or ICD-10 code corresponding to disease/ event in the 1 year prior to LCI episode constituted history of disease/ event. ^§^For each cardiopulmonary event of interest (AMI, ischemic stroke, hemorrhagic stroke, unstable angina) history of that particular event was assessed. | | | | | | | | | | | | |
|  |  |  |  |  |  |  |  |  |  |  |  |  |
|  |  |  |  |  |  |  |  |  |  |  |  |  |
