# Supplementary material for: Coronary and Cerebrovascular Events and Exacerbation of Existing Conditions After Laboratory‐Confirmed Influenza Infection Among US Veterans: A Self‐Controlled Case Series Study
Source: Influenza Other Respir Viruses. 2024 Jun 6;18(6):e13304. doi: 10.1111/irv.13304 (PMC11157146; doi:10.1111/irv.13304)
Supplement: Supplementary file 4 — Table S3. Risk of coronary and cerebrovascular event associated with LCI, sensitivity analysis of risk period. [file IRV-18-e13304-s001.docx]

**Appendix Table 3.** Risk of coronary and cerebrovascular event associated with LCI, sensitivity analysis of risk period

|  | Number episodes | Number unique individuals | Episodes with event during risk interval | Episodes with event during control interval^†^ | IR (95%CI) |  |
| --- | --- | --- | --- | --- | --- | --- |
|  |  |  |  |  |  |  |
| Risk interval days 8-14 after LCI |  |  |  |  |  |  |
|  |  |  |  |  |  |  |
| LCI July 1, 2010- June 30, 2018, severe exacerbation event identified by ICD-9 or ICD-10 codes | | | | |  |  |
| AMI | 2,012 | 1,949 | 38 | 1,974 | 2 (1.4, 2.7) |  |
| Ischemic stroke | 1,167 | 1,138 | 32 | 1,135 | 2.9 (2.1, 4.1) |  |
| Hemorrhagic stroke/ major bleed | 182 | 177 | 0 | 182 | 0 (0, 0) |  |
| Unstable angina/ coronary spasm | 410 | 395 | NR | NR | 0.5 (0.1, 2) |  |
|  |  |  |  |  |  |  |
| Cardiopulmonary event identified by ICD-10 codes only^‡^ | |  |  |  |  |  |
| STEMI | 166 | 164 | NR | NR | 1.3 (0.3, 5.1) |  |
| NSTEMI | 1,090 | 1,058 | 22 | 1,068 | 2.1 (1.4, 3.2) |  |
|  |  |  |  |  |  |  |
| Risk interval days 15-21 after LCI |  |  |  |  |  |  |
|  |  |  |  |  |  |  |
| LCI July 1, 2010- June 30, 2018, severe exacerbation event identified by ICD-9 or ICD-10 codes | | | | |  |  |
| AMI | 1,974 | 1,915 | 38 | 1,936 | 1.8 (1.3, 2.4) |  |
| Ischemic stroke | 1,135 | 1,106 | 27 | 1,108 | 2.2 (1.5, 3.2) |  |
| Hemorrhagic stroke/ major bleed | 182 | 177 | NR | NR | 3.1 (1.4, 6.9) |  |
| Unstable angina/ coronary spasm | 408 | 393 | NR | NR | 1.1 (0.5, 2.7) |  |
|  |  |  |  |  |  |  |
| Cardiopulmonary event identified by ICD-10 codes only^‡^ | |  |  |  |  |  |
| STEMI | 164 | 162 | NR | NR | 0.6 (0.1, 4) |  |
| NSTEMI | 1,068 | 1,039 | 17 | 1,051 | 1.5 (0.9, 2.4) |  |
|  |  |  |  |  |  |  |
| Risk interval days 1-14 after LCI |  |  |  |  |  |  |
|  |  |  |  |  |  |  |
| LCI July 1, 2010- June 30, 2018, severe exacerbation event identified by ICD-9 or ICD-10 codes | | | | |  |  |
| AMI | 2,148 | 2,078 | 174 | 1,974 | 4.5 (3.9, 5.3) |  |
| Ischemic stroke | 1,212 | 1,182 | 77 | 1,135 | 3.5 (2.8, 4.4) |  |
| Hemorrhagic stroke/ major bleed | 193 | 187 | 11 | 182 | 3.1 (1.7, 5.7) |  |
| Unstable angina/ coronary spasm | 415 | 400 | NR | NR | 0.9 (0.4, 1.9) |  |
|  |  |  |  |  |  |  |
| Cardiopulmonary event identified by ICD-10 codes only^‡†^ | |  |  |  |  |  |
| STEMI | 167 | 165 | NR | NR | 0.9 (0.3, 2.9) |  |
| NSTEMI | 1,167 | 1,133 | 99 | 1,068 | 4.7 (3.9, 5.8) |  |
|  |  |  |  |  |  |  |
| Risk interval days 1-21 after LCI |  |  |  |  |  |  |
| LCI July 1, 2010- June 30, 2018, severe exacerbation event identified by ICD-9 or ICD-10 codes | | | | |  |  |
| AMI | 2,148 | 2,078 | 205 | 1,943 | 3.6 (3.1, 4.1) |  |
| Ischemic stroke | 1,212 | 1,182 | 102 | 1,110 | 3.1 (2.5, 3.8) |  |
| Hemorrhagic stroke/ major bleed | 193 | 187 | 17 | 176 | 3.3 (2, 5.4) |  |
| Unstable angina/ coronary spasm | 415 | 400 | 11 | 404 | 0.9 (0.5, 1.7) |  |
|  |  |  |  |  |  |  |
| Cardiopulmonary event identified by ICD-10 codes only^‡^ | |  |  |  |  |  |
| STEMI | 167 | 165 | NR | NR | 0.6 (0.2, 1.9) |  |
| NSTEMI | 1,167 | 1,133 | 114 | 1,053 | 3.7 (3, 4.4) |  |
|  |  |  |  |  |  |  |
| Risk interval days 1-7 after LCI, with 7 day buffer^§^ | |  |  |  |  |  |
| LCI July 1, 2010- June 30, 2018, severe exacerbation event identified by ICD-9 or ICD-10 codes | | | | |  |  |
| AMI | 2133 | 2065 | 136 | 1997 | 7 (5.9, 8.4) |  |
| Ischemic stroke | 1204 | 1174 | 45 | 1159 | 4 (3, 5.4) |  |
| Hemorrhagic stroke/ major bleed | 193 | 187 | 11 | 182 | 6.2 (3.4, 11.5) |  |
| Unstable angina/ coronary spasm | 411 | 396 | NR | NR | 1.3 (0.5, 3.1) |  |
|  |  |  |  |  |  |  |
| Cardiopulmonary event identified by ICD-10 codes only^‡^ | |  |  |  |  |  |
| STEMI | 167 | 165 | NR | NR | 0.6 (0.1, 4.4) |  |
| NSTEMI | 1,157 | 11,23 | 77 | 1,080 | 7.4 (5.8, 9.3) |  |
| **Abbreviations**: CHF, congestive heart failure; COPD, chronic obstructive pulmonary disease; IR, incidence ratio; LCI, laboratory-confirmed influenza. | | | | | |  |
|  |  |  |  |  |  |  |
|  |  |  |  |  |  |  |
| ^†^ Relative to LCI (where day=0), the control interval includes days -365 to 365, excluding days 1-7 and days in the risk interval. | | | | | |  |
| ^‡^ The motivation for looking at ICD-10 codes only is that the ICD-9 codes cannot distinguish these events sufficiently. | | | | | |  |
| ^§^ A buffer period on days 1-7 prior to LCI was included in this analysis; these days were excluded from the risk and control intervals. | | | | | |  |
